# A novel bioactive osteogenesis scaffold delivers ascorbic acid, β-glycerophosphate, and dexamethasone *in vivo* to promote bone regeneration

**DOI:** 10.18632/oncotarget.15779

**Published:** 2017-02-28

**Authors:** Chao Wang, Xuecheng Cao, Yongxian Zhang

**Affiliations:** ^1^ Department of Orthopedic Injury, General Hospital of Jinan Military Area, Tianqiao District, Jinan, Shangdong, China

**Keywords:** osteogenesis scaffold, ascorbic acid, β-glycerophosphate, dexamethasone, bone regeneration

## Abstract

Ascorbic acid, β-glycerophosphate, and dexamethasone have been used in osteogenesis differentiation medium for *in vitro* cell culture, nothing is known for delivering these three bioactive compounds *in vivo*. In this study, we synthesized a novel bioactive scaffold by combining these three compounds with a lysine diisocyanate-based polyurethane. These bioactive compounds were released from the scaffold during the degradation process. The cell culture showed that the sponge-like structure in the scaffold was critical in providing a large surface area to support cell growth and all degradation products of the polymer were non-toxic. This bioactive scaffold enhanced the bone regeneration as evidenced by increasing the expression of three bone-related genes including collagen type I, Runx-2 and osteocalcin in rabbit bone marrow stem cells (BMSCs) *in vitro* and *in vivo*. The osteogenesis differentiation of BMSCs cultured in this bioactive scaffold was similar to that in osteogenesis differentiation medium and more extensive in this bioactive scaffold compared to the scaffold without these three bioactive compounds. These results indicated that the scaffold containing three bioactive compounds was a good osteogenesis differentiation promoter to enhance the osteogenesis differentiation and new bone formation *in vivo*.

## INTRODUCTION

Bone defects are commonly caused by injury, trauma, infection or cancer and a large bone defect is difficult to repair. Bone tissue engineering provides an alternative to bone regeneration and most common models of engineering new tissue are based on seeding isolated cells on a three-dimensional scaffold followed by *in vitro* culture [1].

Polyurethanes are one of the most popular biomaterials for medical applications due to their segmented block copolymeric character. The properties of polyurethanes can be modified by changing the ratio of hard and soft segments and by varying chemical composition [2]. They can be strong elastomers or rigid plastics, and they can be processed using extrusion, injection molding, film blowing, solution dipping, and two-part liquid molding.

Isocyanates are the fundamental starting materials for the synthesis of polyurethanes. Some researchers have even developed a urethane polymer for drug delivery [3], vascular grafts [4], artificial hearts [5] and cartilage regeneration [6, 7]. However, commercial isocyanates are toxic due to their degradation products such as aromatic diamines [8, 9].

Ascorbic acid has shown to promote osteoblast differentiation [10] *in vitro*. β-glycerophosphate is a protein phosphatase inhibitor that acts as a phosphate group donor in matrix mineralization studies [11], [12]. Dexamethasone, a synthetic and widely used glucocorticoid, has the affect of enhancing osteoblast differentiation [13]. These are three important supplements routinely used in osteoblast tissue culture [14]. However, how to deliver these three compounds to the bone defect location and enhance the bone regeneration *in vivo* is still a big challenge in clinics.

In the present study, we have developed a novel, non-toxic, amino acid-based, ascorbic acid, dexamethasone and β-glycerophosphate-containing polyurethane scaffold for bone regeneration. This polyurethane scaffold continues to release these three compounds and keeps high concentrations of these three compounds to enhance the bone formation *in vitro* and *in vivo*.

## RESULTS

**Figure 1:**
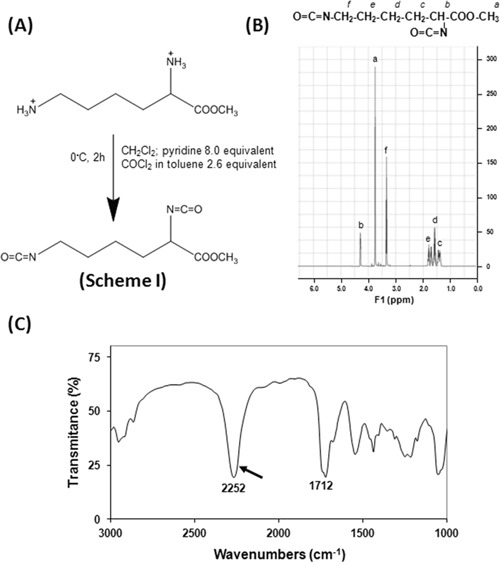
The synthesis of lysine diisocyanate (LDI) methyl ester The LDI was synthesized using lysine methyl ester and phosgene according to the Scheme I **(A)**. FT-IR spectrum showed a strong peak at 2252 cm-1 indicating the product contains isocyanate group **(C)**. Furthermore, ^1^HNMR spectrum demonstrated that a high-quality LDI has been synthesized **(B)**.

Lysine diisocyanate (LDI) is an amino acid-based starting material for polyurethane synthesis and it is not a commercial product currently. In the present study, we have modified the previous method [15] to synthesize LDI from lysine methyl ester according to Scheme I (Figure 1A). The structure of LDI was also consistent with ^1^HNMR spectrum. As shown in Figure 1B: OCN-CH_2_-, δ=3.34, 2H; OCN-CH-C(O), δ=4.25, 1H; C(O)-O- CH_3_, δ =3.75, 3H; -CH_2_-, δ =1.38-1.81, 6H. The characteristic of isocyanate group was exhibited by FT-IR spectrum as evidenced by a strong peak at 2252 cm^−1^ (Figure 1C, arrow). These results indicated that the LDI synthesized with this procedure was of adequate yield and purity for the preparation of LDI-based polymers with potential use in tissue engineering.

**Figure 2:**
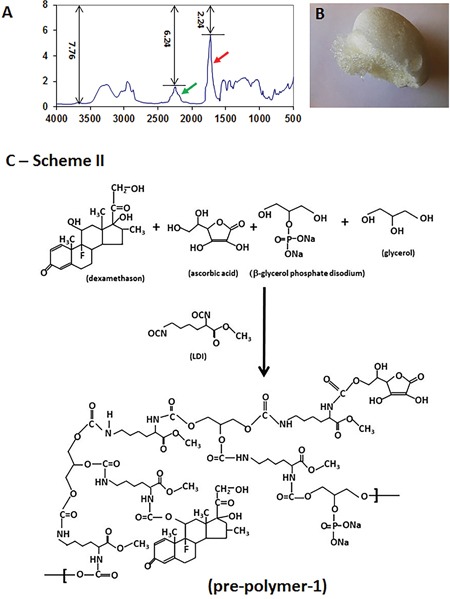
Characterization of LDI-glycerol polymer scaffold The characterization of LDI-glycerol polymer was tested by FT-IR spectrum **(A)**, and image of LDI-glycerol polymer scaffold **(B)**. A sponge-like polymer foam **(B)** was obtained by adding water into reaction mixture when 15% of isocyanate group left in the reaction solution mixture (**A**, arrow). Scheme II shows the synthesis of three bioactive molecules containing pre-polymer-1 using lysine diisocyanate (LDI) methyl ester, glycerol, ascorbic acid, β-glycerophosphate, and dexamethasone at room temperature for 7 days **(C)**.

A polymer scaffold without bioactive molecules was synthesized using LDI and glycerol in a ratio of 1.15:1 (-NCO/−OH) at room temperature without any additional solvent. The urethane formation was demonstrated by a strong peak which appeared at 1724 cm^−1^ in FT-IR spectrum (Figure 2A, red arrow). The reaction was monitored according to the published calculation method [15]: the absorbance of urethane at 1724 cm^−1^ (-NHCOO-) = log 7.76/2.24 = 0.5396; the absorbance of the isocyanate at 2221 cm^−1^ (-NCO) = log 7.76/6.24 = 0.0947; if the concentration of the polyurethane is C1, and that of isocyanate is C2, then C1/C2 = 0.5396/0.09468 = 5.6995. In the reaction mixture, C1 + C2 = 1, thus, 5.6995C2+C2 =1; C2=1/6.6995= 14.93%, and C1 = 85.07%. When the peak at 2221 cm^−1^ (Figure 2A, green arrow) was decreased by 85% (15% left) during the reaction, 0.6 ml of water were added into the reaction mixture and stirred for 30 minutes and a white, sponge-like polymer foam was obtained (Figure 2B).

**Figure 3:**
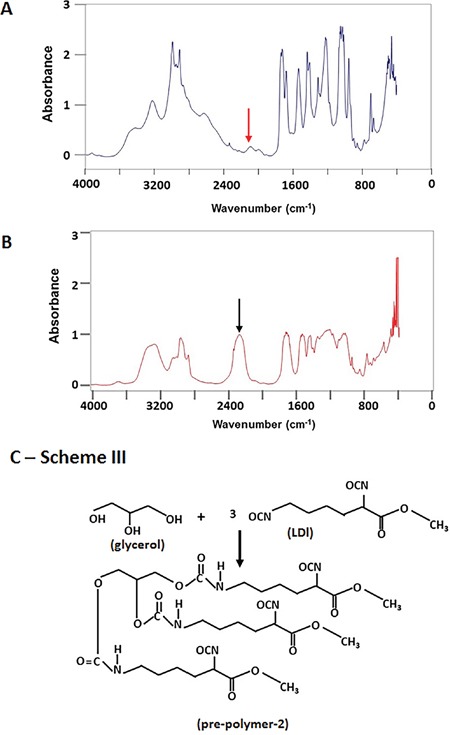
The synthesis of bioactive molecules containing polymer-1 The bioactive compounds containing polymer-1 was monitored by FT-IR spectrum and the reaction was stopped when the peak at 2234 cm-1 disappeared completely (**A**, red arrow). Similarly, an isocyanate-terminated pre-polymer-2 was made by the reaction of LDI and glycerol at a ratio of 2:1 (-NCO/−OH) according to the Scheme III **(C)**. The reaction was stopped when 50% of the isocyanate peak at 2234 cm^−1^ disappeared (50% left, **B**, black arrow).

**Figure 4:**
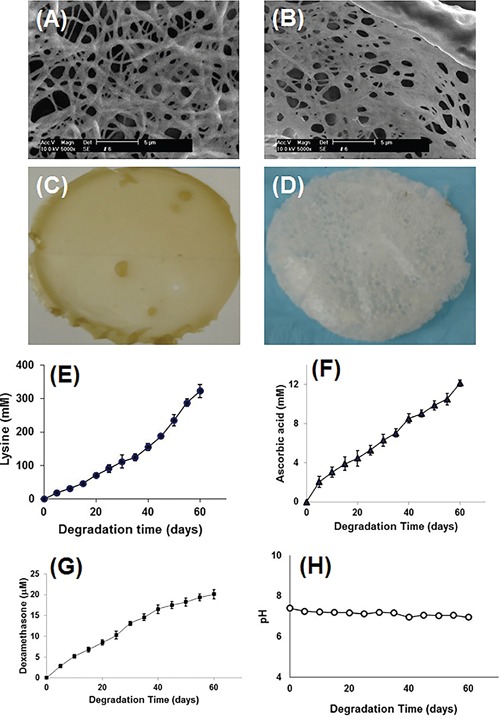
Characterization of bioactive compounds containing scaffold Scanning electron micrograph (SEM) and photo images showed both scaffolds made either by LDI, glycerol, ascorbic acid, β-glycerophosphate, and dexamethasone **(A, C)** or by LDI and glycerol polymer **(B, D)** have a sponge-like structure with the pore size range between 40 and 2500 nm in diameter. The bioactive compounds-containing (LDI-AA-GP-DEX) polymer turned yellow **(C)**, however, the LDI-glycerol polymer remained white after a heat treatment at 100°C for 3 hours **(D)** Bioactive compounds were released from bioactive compounds-containing (LDI-glycerol-AA-GP-DEX) scaffold during the degradation. The degradation products of lysine **(E)**, ascorbic acid **(F)**, and dexamethasone **(G)** were determined and the products did not change the pH of the solution during the degradation **(H)**.

On the other hand, the pre-polymer containing three bioactive molecules was synthesized by adding ascorbic acid (AA), dexamethasone (DEX) and β-glycerophosphate (GP) into LDI and glycerol mixture at a ratio of 1:1 (-OH/NCO) according to the Scheme II (Figure 2C). FT-IR spectrum demonstrated the reaction has been completed and a bioactive molecule-containing pre-polymer has been obtained when the peak at 2234 cm^−1^ disappeared completely (Figure 3A, red arrow) and the product was named as pre-polymer-1 (Figure 2C). Similarly, isocyanate-terminated pre-polymer-2 was made in a ratio of 2:1 (-NCO/−OH) according to the Scheme III (Figure 3C). The reaction was stopped when the FT-IR spectrum showed that the isocyanate peak at 2234 cm^−1^ was decreased to 50% (Figure 3B, black arrow). Finally, the addition of 0.1 ml of β-glycerophosphate-water solution (0.1 g/ml) into a mixture of 0.5 g pre-polymer-1 and 0.5 g pre-polymer-2 resulted in a bioactive compound-containing (LDI-glycerol-AA-GP-DEX) polyurethane scaffold (Figures 4A, 4C).

The scanning electron micrograph (SEM) showed cross-link of the polymer foam made either by bioactive compounds-containing (LDI-glycerol-AA-GP-DEX) polymer (Figure 4A) or by LDI-glycerol (Figure 4B) due to liberation of CO_2_ during the polymerization process. A sponge-like structure was found in the cross-sectional view of the polymer with the pore sizes ranging between 40 to 2500 nm in diameter (Figure 4A and 4B). These pores were interconnected which allows nutrients and other fluids to flow to aide cell migration. The scaffold made by three bioactive compounds-containing (LDI-glycerol-AA-GP-DEX) polymer changed color from white to yellow after 3 hours of heating at 100°C due to ascorbic acid being oxidized (Figure 4C). However, there was no color change found in the scaffold made by LDI-glycerol polymer only even after 3-hours of heating at 100°C (Figure 4D).

The degradation test was performed by adding three bioactive compounds-containing (LDI-glycerol-AA-GP-DEX) polymer scaffold in PBS and measuring the degradation products in the solution for 60 days. As shown in Figure 4, about 83% of the lysine was liberated from the polymer foam during a 2 month degradation (Figure 4E). Both ascorbic acid and dexamethasone were detected in PBS and released at similar rate from the polymer foam (Figure 4F and 4G). The degradation products did not significantly affect the pH over a period of 60 days (Figure 4H). The results indicated that the polymer was degradable and the degradation products were produced by hydrolysis (Scheme IV).

**Scheme IV:**
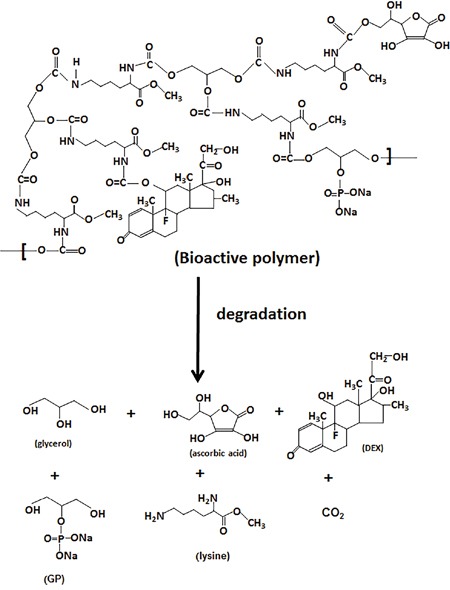
Characterization of bioactive compounds containing scaffold Degradation of bioactive molecules containing polymer scaffold. Non-toxic products were released from the bioactive molecules containing (LDI-glycerol-AA-GP-DEX) polymer scaffold during the degradation.

**Figure 5:**
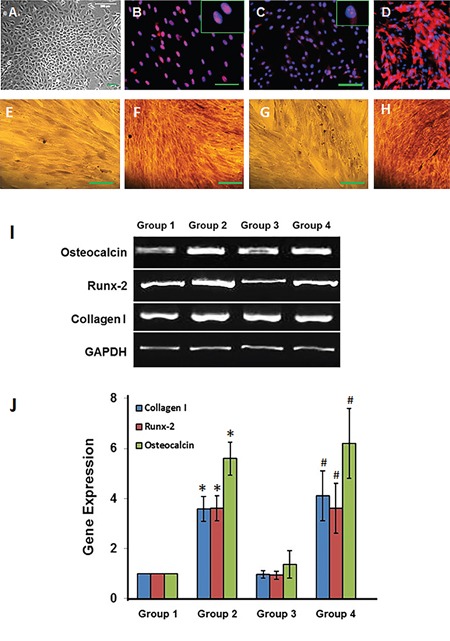
Osteogenesis differentiation of rabbit BMSCs *in vitro* **(A)** A typical colony was formed in the primary culture of rabbit BMCs. **(B)** More than 90% of BMCs expressed Oct-4; **(C)** More than 95% of BMCs presented nucleostemin; (**D**) more than 80% of BMCs were positively stained by SSEA-4. Alizarin Red S staining showed that less than 20% of BMCs either cultured in tissue culture plate with normal medium (Group-1, **E**) or LDI-glycerol scaffold with normal medium (Group-3, **G**) were positively stained by Alizarin Red S. However, more than 80% of BMCs either cultured in tissue culture plate with osteogenesis medium (Group-2, **F**) or in bioactive molecules containing (LDI-glycerol-AA-GP-DEX) polymer scaffold with normal culture medium (Group-4, **H**) were positively stained by Alizarin red S (Bars: 100 μm). Both regular RT-PCR **(I)** and qRT-PCR **(J)** showed that three osteogenesis gene markers, collagen I, Runx-2, and osteocalcin were increased in rabbit BMSCs cultured either in osteogenesis medium (Group 2) or bioactive molecules containing polymer scaffold (Group 4) compared to the BMSCs cultured in tissue culture plate with normal medium (Group 1) and LDI-glycerol polymer scaffold (Group 3). *p<0.05 compared to Group 1; #p<0.05 compared to Group 3.

Rabbit bone marrow stem cells (BMSCs) were used for *in vitro* experiments and immunostaining results showed that the BMSCs isolated from rabbit bone marrow kept high “stemness” as evidenced by colony formation (Figure 5A) and stem cell marker expressions including Oct-4 (Figure 5B), nucleostemin (Figure 5C) and SSEA-4 (Figure 5D). After the BMSCs were cultured under four different conditions for 21 days, the osteogenesis differentiation of these cells was tested by Alizarin Red S (Figures 5E-5H) and regular RT-PCR (Figure 5I) and quantitative real-time RT-PCR (Figure 5J). The histochemical staining indicated that more than 80% rabbit BMSCs cultured in tissue culture plate with osteogenesis medium (Group-2) have differentiated into osteocytes, as shown by staining of calcium deposits (Figure 5F), and similar results were found in the BMSCs cultured in three bioactive compounds-containing (LDI-glycerol-AA-GP-DEX) polymer foam with basic growth medium (Group-4; Figure 5H). However, less than 20% of cells underwent osteogenic differentiation when the BMSCs were cultured in tissue culture plate with basic growth medium (Group-1; Figure 5E). Furthermore, very few cells cultured in LDI-glycerol only polymer foam with basic growth medium (Group-3) were positive stained by Alizarin Red S (Figure 5G).

RT-PCR results showed that three osteogenesis gene markers, collagen type I, Runx-2 and osteocalcin were significantly enhanced in BMSCs cultured in group-2 and group-4 when compare to the Group-1 and Group-3 (Figure 5I and 5J). The gene expression on collagen type I and Runx-2 was more than 3.5 times higher in Groups 2 and 4 than those of Groups-1 and 3. Similarly, the gene expression on osteocalcin was more than 5 times stronger in Groups-2 and 4 than that of Groups-1 and 3 (Figure 5I and 5J).

**Figure 6:**
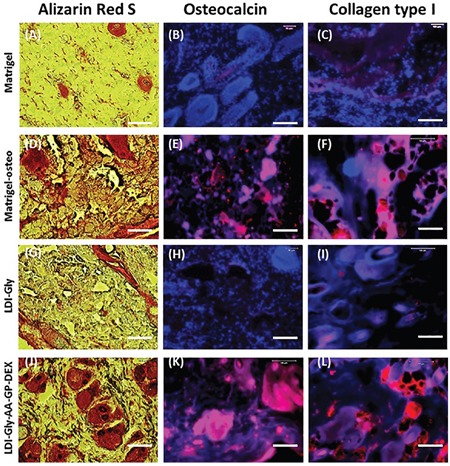
Histological analysis of nude rat skin sections after implantation of rabbit BMSCs with four different scaffolds for 3 weeks *in vivo* (**A-C**) BMSCs in Matrigel with normal medium (Group-1). (**D-F**) BMSCs in Matrigel with osteogenesis medium (Group-2). (**G-I**) BMSCs in LDI-glycerol scaffold with normal medium (Group-3). (**J-L**) are BMSCs in bioactive compounds-containing (LDI-glycerol-AA-GP-DEX) scaffold with normal medium (Group-4). Alizarin Red S staining shows that BMSCs grown in bioactive compounds-containing (LDI-glycerol-AA-GP-DEX) polymer scaffold have differentiated into osteocytes **(J)** and these cells were positively stained by osteocalcin **(K)** and collagen type I **(L)**. Similarly, the BMSCs grown in Matrigel with osteogenesis medium also positively stained by Alizarin red S **(D)**, osteocalcin **(E)** and collagen type I **(F)**. On the other hand, very few BMSCs were positively stained by these markers when they grew either in LDI-glycerol scaffold **(G-I)** or Matrigel **(A-C)** with normal medium. Bars: 100 μm.

To determine whether the three bioactive compounds-containing (LDI-glycerol-AA-GP-DEX) polymer scaffold treated rabbit BMSCs underwent osteogenesis differentiation *in vivo*, rabbit BMSCs cultured with three bioactive compounds-containing (LDI-glycerol-AA-GP-DEX) polymer scaffold were implanted into nude rats subcutaneously. Alizarin red S staining showed that BMSCs grown in three bioactive compounds-containing (LDI-glycerol-AA-GP-DEX) polymer scaffold have differentiated into osteocytes (Figure 6J) and these cells were positively stained by osteocalcin (Figure 6K) and collagen type I (Figure 6L). Similarly, the BMSCs grown in Matrigel with osteogenesis medium also positively stained by Alizarin Red S (Figure 6D), osteocalcin (Figure 6E) and collagen type I (Figure 6F). On the other hand, very few BMSCs were positively stained by these markers when they grew either in LDI-glycerol scaffold (Figures 6G-6I) or Matrigel (Figures 6A-6C) with normal medium. Both histological-chemical staining and immunostaining indicated that after three weeks implantation, bone-like tissue was extensively formed in three bioactive compounds-containing (LDI-glycerol-AA-GP-DEX) polymer scaffold group, however, in control groups (BMSCs in Matrigel or in LDI-glycerol polymer scaffold with normal growth medium) formed little bone-like tissue (Figure 6).

## DISCUSSION

Polyurethane scaffolds display excellent mechanical properties and are widely used in medical implantations. However, many medical-grade polyurethanes are prepared from aromatic diisocyanates and degradation products *in vivo* are carcinogenic aromatic diamines. It is therefore desirable to synthesize new medical-grade polyurethanes from nontoxic aliphatic diisocyanates. Lysine diisocyanate (LDI) is an amino acid-based starting material for polyurethane synthesis; unfortunately, it is not a commercial product currently.

In this study, a high-quality LDI has been synthesized according to previous protocol described by Zhang *et al*. [15]. By using this amino acid-based LDI to react with glycerol, a non-toxic polyurethane backbone was synthesized. With the addition of ascorbic acid, β-glycerophosphate and dexamethasone into this LDI-glycerol based polyurethane backbone; a three bioactive molecule-containing polyurethane scaffold was developed for bone tissue engineering. *In vitro* and *in vivo* experimental results indicated that this bioactive polyurethane scaffold is biodegradable and biocompatible. The osteogenesis differentiation of rabbit BMSCs grown in this bioactive polyurethane scaffold with normal growth medium was similar to that grown in tissue culture plate with osteogenesis medium and much stronger than those grown in tissue culture plate or LDI-Glycerol scaffold with normal medium.

Mature bone is composed of proteins and minerals. Approximately 90% the matrix proteins in bone are collagen. Ascorbic acid was found to stimulate cell proliferation [16, 17]., enhance the collagen synthesis [18], activate alkaline phosphatase [19], and induce the bone cell differentiation [20]. Collagenase is a destructive enzyme secreted by cells to degrade the extracellular matrix protein, collagen, thus weakening the primary support structures of the skin and bone. Ascorbic acid is a potent inhibitor of collagenase and the addition of ascorbic acid into culture medium will enhance the collagen synthesis.

β-glycerophosphate is a simple phosphate donor and a classical serine-threonine phosphatase inhibitor used in kinase reaction buffer [12]. Ascorbic acid, in association with β-glycerophosphate, was found to stimulate matrix mineralization by inducing an increase of neutral metalloproteinase in matrix vesicles, which may be able to degrade proteoglycans favoring mineral precipitation [21].

Dexamethasone is a parathyroid hormone which significantly induced the differentiation of osteoblastic cells. Human alveolar bone cells grown in the continuous presence of dexamethasone presented an earlier appearance and higher levels of alkaline phosphatase, and an increased ability to form calcium phosphate deposits in the extracellular matrix [22].

The effect of ascorbic acid, β-glycerophosphate and dexamethasone on osteoblastic differentiation has been investigated [23, 24]. It has been reported that the presence of dexamethasone, either alone or in combination, resulted in an evident increase in cell growth. The studies have demonstrated that ascorbic acid, β-glycerophosphate, and dexamethasone induced an increase of the mRNA level for collagen type I, osteocalcin, bone sialoprotein, and alkaline phosphatase in association with the development of bone nodules in an *in vitro* system [14, 25].

An *in vitro* experiment indicated that β-glycerophosphate promoted mineralization and almost 80% of 10 mM β-glycerophosphate was hydrolyzed by bone cells within 24 hours [12]. In the present study, we combined these three bioactive compounds to LDI-Glycerol polyurethane scaffold and added additional β-glycerophosphate into the foam by dissolving β-glycerophosphate into water when the foam formation, therefore the concentrations of β-glycerophosphate, as well as ascorbic acid and dexamethasone were kept at higher level during the polymer degradation.

A novel osteogenesis supplement polyurethane scaffold has been synthesized; the *in vitro* and *in vivo* results indicate that this ascorbic acid, β-glycerophosphate, and dexamethasone-containing polyurethane scaffold enhanced bone formation and can be used for bone tissue engineering.

## MATERIALS AND METHODS

### Materials

Ascorbic acid, dexamethasone, glycerol, glycerol-2-phosphate disodium salt, lysine methyl ester dihydrochloride and all other reagents were analytical grade and obtained from Sigma-Aldrich (St. Louis, MO, USA) unless otherwise stated.

### Preparation of bioactive compounds-containing pre-polymer

In order to prepare bioactive compounds-containing prepolymer, we first synthesized and purified lysine diisocyanate (LDI) according to the published protocol with some modification [15]. Then, we used the LDI and glycerol to synthesize the bioactive reagents-containing pre-polymer using the previous protocol [15].

In a typical experiment, 80 mg glycerol-2-phosphate disodium salt (MW 216, 370 μmol), 200 mg ascorbic acid (MW 176.12, 1.14 mmol), 1.0 mg dexamethasone (MW 392.46, 2.548 μmol) and 1 ml glycerol (MW 92, 13.696 mmol, -OH 41.09 mmol) were added into 2 ml of dimethyl sulfoxide (DMSO) in a dry round-bottomed flask, flushed with nitrogen, fitted with rubber septa and sealed with paraffin film. The mixture was continuously stirred until all three bioactive reagents dissolved. Subsequently, 3.8 ml LDI (MW 212, 20.72 mmol, -NCO 41.44 mmol) were added by a syringe and the reaction was performed by continuously stirring at room temperature in the dark for 7 days. The reaction was monitored by Fourier transform infrared (FT-IR) spectroscopy and stopped by the addition of diethyl ether when the FT-IR spectrum showed that the isocyanate peak completely disappeared. At this point, the pre-polymer was precipitated as a white solid and named prepolymer-1 which was either re-dissolved with CH_2_Cl_2_ and used immediately or stored at 4°C until use.

### Preparation of LDI-glycerol polymer foam

The LDI-glycerol polymer foam was synthesized by the similar procedures as prepolymer-1 without adding the three bioactive compounds and controlled the ratio of the function groups (–NCO/−OH) at 1.15:1. The reaction was monitored by FT-IR spectroscopy and stopped when the FT-IR spectrum showed about a 15% isocyanate peak left in the reaction mixture. At this point, 0.6 ml water was added and stirred for 30 minutes to form the foam.

### Preparation of isocyanate terminated prepolymer

The isocyanate-terminated pre-polymer was prepared by reacting LDI and glycerol at the ratio of 2:1 (–NCO/−OH) and following the similar protocol described above. The reaction was stopped when the FT-IR spectrum showed the isocyanate peak has decreased to 50%. At that time a viscous solution was obtained and named prepolymer-2 which was used immediately or stored at 4°C until use.

### Preparation of bioactive compounds-containing polyurethane scaffold

Finally, the prepolymer-1 and prepolymer-2 were mixed together (1:1 by weight) and a β-glycerophosphate (GP) solution (100 mg glycerol-2-phosphate disodium salt was dissolved in 1 ml water) was used to crosslink these two pre-polymers and generate a new three bioactive reagent-containing (LDI-glycerol-AA-GP-DEX) polymer foam. Typically, 100 μl of GP solution was added to 1 g of the mixture of pre-polymers (0.5 g prepolymer-1 + 0.5 g prepolymer-2) at room temperature and stirred for 30 minutes. The polymer foam was enhanced and dried at 40°C overnight in a vacuum oven.

### Pore size distribution of polymer foam

The pore size distribution of the polymer foam was measured using scanning electron micrograph (SEM) according to the published protocol [26]. The pictures of polymer scaffold were obtained from three pieces of each polymer foam and the pore size distribution of the polymer foam was determined using Imaging J.

### Bioactive compounds distribution of polymer foam

The distribution of bioactive reagents in the bioactive compounds-containing (LDI-glycerol-AA-GP-DEX) polymer foam was tested by heating the polymer foam at 100°C for 3 hours; ascorbic acid distribution was indicated by the appearance of yellow color in the bioactive compounds-containing (LDI-glycerol-AA-GP-DEX) polymer foam. LDI-glycerol polymer foam was used as a control and treated the same as the bioactive compounds-containing polymer foam. Three random pieces from each polymer scaffold were tested.

### Degradation of bioactive compounds-containing polymer scaffold *in vitro*

Each 100 mg of polymer scaffold was cut into small pieces (1 mm × 1 mm × 1 mm) and put in a centrifuge tube with 1 ml of phosphate-buffered solution (PBS). The mixture of the scaffold and PBS was mixed well using a vortex mixer and then incubated at 37°C for 1-60 days. The degradation products released from the polymer scaffold were detected in the supernatant collected every day by centrifuging the tube at 10 000 g for 10 min. After transferring the supernatant to a new tube, 1 ml of new PBS was added into the pellet (scaffold) and mixed well using a vortex mixer. The PBS was changed every day by repeating the above procedures. The concentration of lysine released from the polymer was determined by ninhydrin colorimetric reaction according to the published protocol [27]. Each sample was assayed in triplicate and the average was used to determine the final lysine content.

The concentration of ascorbic acid liberated from the bioactive compounds-containing (LDI-glycerol-AA-GP-DEX) polymer foam was measured by an ascorbic acid assay kit according to the manufacturer's protocol (BioVision, Cat. #K661-100). For the determination of the concentration of dexamethasone the published method was used [28] and three samples at each time point were tested during the degradation.

### Isolation and culture of bone marrow stem cells (BMSC)

BMSCs were obtained from femur bone marrow of 12-15 weeks old New Zealand white rabbits. The protocol for animal use in this study was approved by IACUC of General Hospital of Jinan Military Area. Briefly, a femur bone was washed with PBS plus penicillin (100 U/ml) and streptomycin (100 μg/ml). Subsequently, its’ metaphyses were removed and the marrow flushed from the bone with 5 ml PBS containing 10 units/ml heparin. The cells were harvested into a centrifuge tube and washed twice by centrifugation at 1 100 g for 5 minutes. The cell pellet was cultured in DMEM with 20% FBS containing 5 units/ml heparin, 1% penicillin (working concentration 100 U/ml) and streptomycin (working concentration 100 μg/ml) at 37°C and 5% CO_2_. The stemness of BMSCs was tested by immunostaining of nucleostemin, Oct-4, and strol-1, three stem cell markers.

### Osteogenesis of BMSCs cultured with four conditions *in vitro*

BMSCs at passage 1 were seeded in a 6-well plate at a density of 2.4 ×10^5^ cells/well in basic growth medium consisting of low glucose DMEM, 10% heat-inactivated fetal bovine serum (FBS), 100 U/ml of penicillin and 100 μg/ml of streptomycin (Group-1); or with osteogenic induction medium (Millipore, Cat. No. SCR028) consisting of basic growth medium supplemented with 0.1 μM dexamethasone, 0.2 mM ascorbic 2-phosphate, and 10 mM glycerol 2-phosphate (Group-2). The bioactive compounds-containing (LDI-glycerol-AA-GP-DEX) polymer scaffold pieces (200 mg/piece) were washed 5 times each with 75% alcohol, sterile deionized water, and sterile PBS. Then the polymer scaffold was treated under UV light in the tissue culture hood overnight. Each 100 μl of basic growth medium containing 2.4 × 10^5^ BMSCs at passage-1 were seeded in each piece of scaffold in a 6-well plate (one piece/well) and left undisturbed in an incubator for 4 hours to allow the cells to adhere. Subsequently, 1.9 ml of basic growth medium was gradually added to each well (Group-4). The cells were cultured at 37°C, with 5% CO_2_ and 95% air. The same procedures were used for LDI-glycerol scaffold treatment and BMSC culture (Group 3). The culture media were changed every 3 days. The differentiated cells released calcium-rich deposits, which were stained by the Alizarin Red S assay (Millipore, Billerica, MA, Cat. # 2003999) following the manufacturer's protocol. The osteoblasts containing mineral deposits would be stained red or dark brown and analyzed under a microscope [29, 30] (Nikon Instruments, Melville, NY).

### Immunostaining of BMSCs

The “stemness” of rabbit BMSCs were tested by immunocytochemistry. Briefly, cells were fixed with 4% paraformaldehyde in PBS for 30 minutes at room temperature and treated with 2% mouse or goat serum for 30 minutes. For nucleostemin and Oct-4 staining, the cells were treated with 0.1% Triton X-100 for 15 minutes. Then the cells were incubated at room temperature with goat anti-nucleostemin antibody (1:500; Neuromics, Edina, MN, Cat. # GT1505), mouse anti-Oct-4 antibody (1:400; Millipore, Cat. P20263), and mouse anti-SSEA-4 antibody (1:350; Invitrogen, Carlsbad, CA, Cat. # 414000). After 5 hours of reaction time, the primary antibodies were removed and the cells were washed three times with PBS, Cy3-conjugated donkey anti-goat IgG (1:500; Millipore, Cat. # AP180C) for nucleostemin, Cy3-conjugated goat anti-mouse IgG (1:500; Invitrogen, Cat. # A10521) for Oct-4 and SSEA-4 were applied. The cells treated with the same procedures without primary antibodies were used as negative controls, and at least two independent wells were stained by each marker. The stained cells were examined and color images of cells were obtained using a fluorescence microscope.

### Semi-quantification of the extent of cell differentiation

Semi-quantification was used to measure the positive stained cells. Briefly, 12 views from each well were randomly chosen on a microscope with the same magnification (20×). The areas of positive staining were identified manually and measured by SPOT IMAGING Software. The proportion of positive staining was calculated by dividing the stained area by the view area. Twelve ratio values for each of two wells were averaged to obtain the percentage of positive staining, which represents the extent of cell differentiation in the respective induction medium and bioactive compounds-containing (LDI-glycerol-AA-GP-DEX) polymer scaffold.

### Gene expression analysis by real-time RT-PCR

RNA was extracted from BMSCs cultured in four different conditions by using the RNeasy Mini Kit with an on-column DNase I digest (Qiagen, Cat. # 74104). First-strand cDNA was synthesized from 1 μg total RNA in a 20 μl reaction mixture by reverse transcription using SuperScript II (Invitrogen, Cat. # 18064-014). The protocols used for cDNA synthesis were: 65°C for 5 minutes and cooling for 1 minute at 4°C, then 42°C for 50 minutes and 72°C for 15 minutes. qRT-PCR was performed using QIAGEN QuantiTect SYBR Green PCR Kit (Life Technologies, Cat. 4367659). In a 25 μl PCR reaction mixture, 2 μl cDNA (total 100 ng RNA) were amplified in a Chromo 4 Detector (MJ Research, Waltham, MA) with incubation at 94°C for 5 minutes, followed by 50 cycles of a three temperature program of 1 minute at 94°C, 30 seconds at 57°C, and 30 seconds at 72°C. A 5 μl sample of PCR product was taken out at 30 cycles and applied to an agarose gel. The PCR reaction was terminated after 50 cycles with a 10-minute extension at 70°C. The rabbit-specific primers based on previous publications [31, 32] were used: Runx-2, collagen I and osteocalcin. Glyceraldehyde 3-phosphate dehydrogenase (GAPDH) served as an internal control (Table 1). All primers were synthesized by Invitrogen.

**Table 1:** Rabbit primers used for real time RT-PCR

Gene	Type	Primer Sequence	Reference
Collagen I	Forward	5′-CTG ACT GGA AGA GCG GAG AGT AC-3′	Emans PJ et al.
	Reverse	5′-CCA TGT CGC AGA AGA CCT TGA-3′	
Runx-2	Forward	5′-TGA TGA CAC TGC CAC CTC TGA-3′	Emans PJ et al.
	Reverse	5′-GCA CCT GCC TGG CTC TTC T-3′	
Osteocalcin	Forward	5′-GTG CAG AGT CTG GCA GAG G-3′	Alfotawi R, et al.
	Reverse	5′-GGT TGA GCT CGC ACA CCT-3′	
GAPDH	Forward	5′-ACT TTG TGA AGC TCA TTT CCT GGT A-3′	Emans PJ et al.
	Reverse	5′-GTG GTT TGA GGG CTC TTA CTC CTT-3′	

### BMSC differentiation *in vivo*

Eight female nude rats (10 weeks old; 200–250 g) were used to test the effects of bioactive compounds-containing (LDI-glycerol-AA-GP-DEX) polymer scaffold on rabbit BMSC differentiation *in vivo*. Rats were housed individually on a 12 h:12 h light–dark cycle and were cared for in accordance with the Guide to the Care and Use of Experimental Animals.

Rabbit BMSCs at passage 2 (6 ×10^4^/piece/well) were cultured in four conditions: group-1: in Matrigel (0.5 ml/each; Cat. # 354234, BD Biosciences, Bedford, MA) with basic growth medium; group-2: in Matrigel (0.5 ml/each; Cat. # 354234, BD Biosciences, Bedford, MA) with osteogenesis differentiation medium; group-3: in LDI-glycerol scaffold (50 mg/piece) with basic growth medium; group-4: in bioactive compounds-containing (LDI-glycerol-AA-GP-DEX) scaffold (50 mg/piece) with basic growth medium and cultured in a 24-well plate with basic growth medium overnight.

For implantation experiment, the nude rats were placed under general anesthesia using isoflurane. Six wounds of 1 cm diameter each were created on the back of each rat; three wounds per side. The approximate distance between two wound sites was 2 cm. Then one piece of either cell-Matrigel or cell-scaffold was placed in each of three distinct wounds. A total of three cell-Matrigel and three cell-scaffold composites were implanted into each rat. Each group had two rats and a total of eight rats were used for four groups. At 3 weeks after implantation, tissue samples were harvested and placed in pre-labeled base molds filled with frozen section medium (Neg 50; Richard-Allan Scientific; Kalamazoo, MI). The base mold with tissue samples was quickly immersed in liquid nitrogen cold 2-methylbutane and allowed to solidify completely. The tissue blocks were then placed on dry ice and subsequently stored in a deep freezer (-80°C) until used for histological analysis.

### Histochemical and immunohistochemical analyses of tissue sections

The tissue block was cut into 10 μm thick sections, and stained with Alizarin Red S at room temperature for 40 minutes to test bone-like tissue formation. In addition, immunostaining for collagen type I and osteocalcin expression were also performed to verify osteogenesis of BMSCs. Osteocalcin is a noncollagenous protein produced solely by osteoblasts [33]; hence, it was used as a marker of osteogenesis of BMSCs in this study. The tissue sections were reacted either with mouse anti-collagen type I antibody (1:350; Santa Cruz Biotechnology, Inc., Santa Cruz, CA) at room temperature for 3 hours, or mouse anti-osteocalcin antibody (1:350; Abcam, Cambridge, MA) at room temperature for 5 hours. Then the tissue sections were washed 3 times with PBS, reacted with Cy3-conjugated goat anti-mouse IgG (1:500; Santa Cruz Biotechnology, Inc. Santa Cruz, CA) at room temperature for 2 hours, again washed 3 times with PBS, and then reacted with Hoechst fluorochrome 33342 (1 μg/ml; Sigma, St. Louis, MO) at room temperature for 5 minutes. Finally, the sections were washed with PBS twice. Negative control was created by omitting primary antibodies during the immunostaining. The positive stained cells were examined with a fluorescence microscope.

### Statistical analysis

Data presented in this study are the results of three separate experiments performed in cell cultures and mRNA expression. Unless otherwise indicated, data were expressed in mean ± SD. Statistical analyses were conducted using the Microsoft Excel program, one-way ANOVA was used. A *p*-value <0.05 was considered to be significantly different.

## Abbreviations

AA: Ascorbic acid
BMSCs: bone marrow stem cells
DEX: dexamethasone
DMSO: dimethyl sulfoxide
FBS: fetal bovine serum
FT-IR: Fourier transform infrared spectroscopy
GAPDH: Glyceraldehyde 3-phosphate dehydrogenase
GP: β-glycerophosphate
LDI: lysine diisocyanate
PBS: phosphate-buffered solution
qRT-PCR: Real-time quantitative reverse transcription polymerase chain reaction
RT-PCR: Reverse transcription polymerase chain reaction
SEM: scanning electron micrograph

## References

[1] Shinoka T, Breuer CK, Tanel RE, Zund G, Miura T, Ma PX, Langer R, Vacanti JP, Mayer JE (1995). Tissue engineering heart valves: valve leaflet replacement study in a lamb model. Ann Thorac Surg.

[2] Taylor JE, Laity PR, Wong SS, Norris K, Khunkamchoo P, Cable M, Andrews G, Johnson A, Cameron RE (2006). Examination of hard segment and soft segment phase separation in polyurethane medical materials by electron microscopy techniques. Microsc Microanal.

[3] Cherng JY, Hou TY, Shih MF, Talsma H, Hennink WE (2013). Polyurethane-based drug delivery systems. Int J Pharm.

[4] Hong Y, Ye SH, Pelinescu AL, Wagner WR (2012). Synthesis, characterization, and paclitaxel release from a biodegradable, elastomeric, poly(ester urethane)urea bearing phosphorylcholine groups for reduced thrombogenicity. Biomacromolecules.

[5] Silvetti MS, Drago F, Rava L (2012). Long-term outcome of transvenous bipolar atrial leads implanted in children and young adults with congenital heart disease. Europace.

[6] Efe T, Getgood A, Schofer MD, Fuchs-Winkelmann S, Mann D, Paletta JR, Heyse TJ (2012). The safety and short-term efficacy of a novel polyurethane meniscal scaffold for the treatment of segmental medial meniscus deficiency. Knee Surg Sports Traumatol Arthrosc.

[7] Verdonk R, Verdonk P, Huysse W, Forsyth R, Heinrichs EL (2011). Tissue ingrowth after implantation of a novel, biodegradable polyurethane scaffold for treatment of partial meniscal lesions. Am J Sports Med.

[8] Guelcher SA, Gallagher KM, Didier JE, Klinedinst DB, Doctor JS, Goldstein AS, Wilkes GL, Beckman EJ, Hollinger JO (2005). Synthesis of biocompatible segmented polyurethanes from aliphatic diisocyanates and diurea diol chain extenders. Acta Biomater.

[9] Batich C, Williams J, King R (1989). Toxic hydrolysis product from a biodegradable foam implant. J Biomed Mater Res.

[10] Franceschi RT, Iyer BS, Cui Y (1994). Effects of ascorbic acid on collagen matrix formation and osteoblast differentiation in murine MC3T3-E1 cells. J Bone Miner Res.

[11] Shioi A, Nishizawa Y, Jono S, Koyama H, Hosoi M, Morii H (1995). Beta-glycerophosphate accelerates calcification in cultured bovine vascular smooth muscle cells. Arterioscler Thromb Vasc Biol.

[12] Chung CH, Golub EE, Forbes E, Tokuoka T, Shapiro IM (1992). Mechanism of action of beta-glycerophosphate on bone cell mineralization. Calcif Tissue Int.

[13] Ma X, Zhang X, Jia Y, Zu S, Han S, Xiao D, Sun H, Wang Y (2013). Dexamethasone induces osteogenesis via regulation of hedgehog signalling molecules in rat mesenchymal stem cells. Int Orthop.

[14] Park JB (2012). The effects of dexamethasone, ascorbic acid, and beta-glycerophosphate on osteoblastic differentiation by regulating estrogen receptor and osteopontin expression. J Surg Res.

[15] Zhang JY, Beckman EJ, Piesco NP, Agarwal S (2000). A new peptide-based urethane polymer: synthesis, biodegradation, and potential to support cell growth *in vitro*. Biomaterials.

[16] Roach HI, Hillier K, Shearer JR (1985). Ascorbic acid requirements for collagen synthesis (proline hydroxylation) during long-term culture of embryonic chick femurs. Biochim Biophys Acta.

[17] Peterkofsky B (1972). The effect of ascorbic acid on collagen polypeptide synthesis and proline hydroxylation during the growth of cultured fibroblasts. Arch Biochem Biophys.

[18] Berg RA, Steinmann B, Rennard SI, Crystal RG (1983). Ascorbate deficiency results in decreased collagen production: under-hydroxylation of proline leads to increased intracellular degradation. Arch Biochem Biophys.

[19] Choong PF, Martin TJ, Ng KW (1993). Effects of ascorbic acid, calcitriol, and retinoic acid on the differentiation of preosteoblasts. J Orthop Res.

[20] Choi KM, Seo YK, Yoon HH, Song KY, Kwon SY, Lee HS, Park JK (2008). Effect of ascorbic acid on bone marrow-derived mesenchymal stem cell proliferation and differentiation. J Biosci Bioeng.

[21] Coelho MJ, Fernandes MH (2000). Human bone cell cultures in biocompatibility testing. Part II: effect of ascorbic acid, beta-glycerophosphate and dexamethasone on osteoblastic differentiation. Biomaterials.

[22] Adelina Costa M, Helena Fernandes M (2000). Long-term effects of parathyroid hormone, 1,25-dihydroxyvitamin d(3), and dexamethasone on the cell growth and functional activity of human osteogenic alveolar bone cell cultures. Pharmacol Res.

[23] Gupta A, Leong DT, Bai HF, Singh SB, Lim TC, Hutmacher DW (2007). Osteo-maturation of adipose-derived stem cells required the combined action of vitamin D3, beta-glycerophosphate, and ascorbic acid. Biochem Biophys Res Commun.

[24] Dean DD, Schwartz Z, Bonewald L, Muniz OE, Morales S, Gomez R, Brooks BP, Qiao M, Howell DS, Boyan BD (1994). Matrix vesicles produced by osteoblast-like cells in culture become significantly enriched in proteoglycan-degrading metalloproteinases after addition of beta-glycerophosphate and ascorbic acid. Calcif Tissue Int.

[25] Langenbach F, Handschel JR (2013). Effects of dexamethasone, ascorbic acid and beta-glycerophosphate on the osteogenic differentiation of stem cells *in vitro*. Stem Cell Res Ther.

[26] Zhang JY, Beckman EJ, Hu J, Yang GG, Agarwal S, Hollinger JO (2002). Synthesis, biodegradability, and biocompatibility of lysine diisocyanate-glucose polymers. Tissue Eng.

[27] Sivak WN, Pollack IF, Petoud S, Zamboni WC, Zhang J, Beckman EJ (2008). LDI-glycerol polyurethane implants exhibit controlled release of DB-67 and anti-tumor activity *in vitro* against malignant gliomas. Acta Biomater.

[28] Savaliya AA, Prasad B, Raijada DK, Singh S (2009). Detection and characterization of synthetic steroidal and non-steroidal anti-inflammatory drugs in Indian ayurvedic/herbal products using LC-MS/TOF. Drug Test Anal.

[29] Bi Y, Ehirchiou D, Kilts TM, Inkson CA, Embree MC, Sonoyama W, Li L, Lee AJ, Seo BM, Zhang L, Shi S, Young MF (2007). Identification of tendon stem/progenitor cells and the role of the extracellular matrix in their niche. Nat Med.

[30] Zhang J, Wang JH (2010). Characterization of differential properties of rabbit tendon stem cells and tenocytes. BMC Musculoskelet Disord.

[31] Emans PJ, Spaapen F, Surtel DA, Reilly KM, Cremers A, van Rhijn LW, Bulstra SK, Voncken JW, Kuijer R (2007). A novel *in vivo* model to study endochondral bone formation; HIF-1alpha activation and BMP expression. Bone.

[32] Alfotawi R, Naudi K, Dalby MJ, Tanner KE, McMahon JD, Ayoub A (2013). Assessment of cellular viability on calcium sulphate/hydroxyapatite injectable scaffolds. J Tissue Eng.

[33] Puchacz E, Lian JB, Stein GS, Wozney J, Huebner K, Croce C (1989). Chromosomal localization of the human osteocalcin gene. Endocrinology.

